# Role of Extracellular Vesicles and microRNAs on Dysfunctional Angiogenesis during Preeclamptic Pregnancies

**DOI:** 10.3389/fphys.2016.00098

**Published:** 2016-03-18

**Authors:** Carlos A. Escudero, Kurt Herlitz, Felipe Troncoso, Jesenia Acurio, Claudio Aguayo, James M. Roberts, Grace Truong, Gregory Duncombe, Gregory Rice, Carlos Salomon

**Affiliations:** ^1^Group of Investigation in Tumor Angiogenesis, Vascular Physiology Laboratory, Universidad del Bío-BíoChillán, Chile; ^2^Group of Research and Innovation in Vascular Health, Department of Basic Sciences, Universidad del Bío-BíoChillán, Chile; ^3^Department of Clinical Biochemistry and Immunology, Faculty of Pharmacy, University of ConcepciónConcepción, Chile; ^4^Departments of Obstetrics, Gynecology and Reproductive Sciences, Epidemiology, and the Clinical and Translational Science Institute, Magee-Womens Research Institute, University of PittsburghPittsburgh, PA, USA; ^5^Exosome Biology Laboratory, Faculty of Medicine and Biomedical Sciences, Centre for Clinical Diagnostics, UQ Centre for Clinical Research, The University of QueenslandBrisbane, QLD, Australia; ^6^Ochsner Clinic Foundation, Maternal-Fetal Medicine, Department of Obstetrics and GynecologyNew Orleans, LA, USA

**Keywords:** preeclampsia, exosomes, microRNAs, endothelial dysfunction

## Abstract

Preeclampsia is a syndrome characterized by hypertension during pregnancy, which is a leading cause of morbidity and mortality in both mother and newborn in developing countries. Some advances have increased the understanding of pathophysiology of this disease. For example, reduced utero-placental blood flow associated with impaired trophoblast invasion may lead to a hypoxic placenta that releases harmful materials into the maternal and feto-placental circulation and impairs endothelial function. Identification of these harmful materials is one of the hot topics in the literature, since these provide potential biomarkers. Certainty, such knowledge will help us to understand the miscommunication between mother and fetus. In this review we highlight how placental extracellular vesicles and their cargo, such as small RNAs (i.e., microRNAs), might be involved in endothelial dysfunction, and then in the angiogenesis process, during preeclampsia. Currently only a few reports have addressed the potential role of endothelial regulatory miRNA in the impaired angiogenesis in preeclampsia. One of the main limitations in this area is the variability of the analyses performed in the current literature. This includes variability in the size of the particles analyzed, and broad variation in the exosomes considered. The quantity of microRNA targets genes suggest that practically all endothelial cell metabolic functions might be impaired. More studies are required to investigate mechanisms underlying miRNA released from placenta upon endothelial function involved in the angiogenenic process.

## Introduction

Preeclampsia is a syndrome diagnosed with hypertension and concomitant multisystemic dysfunction during pregnancy. Epidemiological and socio-economic studies highlight the relevance of preeclampsia worldwide. However, despite the fact that the pathophysiology of preeclampsia is not entirely understood; there is no doubt that endothelial dysfunction in both maternal and feto-placental circulation is one of the hallmarks in this disease. In this review we propose to highlight new biomarkers, such as extracellular vesicles and small RNAs (i.e., microRNAs) involved in endothelial dysfunction during preeclampsia. Since, abundant information is available in the current literature on this topic, we will restrict our analysis to information regarding angiogenesis as a target.

## Preeclampsia: general overview

Preeclampsia is syndrome characterized by new onset hypertension and proteinuria after 20 weeks of gestation (Roberts and Hubel, 2009). However, the American College of Obstetricians and Gynecologists has stated in their revised guidelines that proteinuria is no longer absolutely required for diagnosis of preeclampsia (ACOG TFoHiP, 2013). In the absence of proteinuria, the diagnosis may be established by the presence of hypertension associated with thrombocytopenia, impaired liver functions, and the development of renal insufficiency, pulmonary edema, or the onset cerebral or visual disturbances previously not experienced.

Preeclampsia affects about 10% of all pregnancies worldwide (Duley, 2009). Globally, it is the leading cause of maternal and neonatal mortality. This imposes substantial burdens on the families of pregnant women, their communities, and healthcare systems (Duley, 2009). Each year, it is estimated that hypertension during pregnancy, particularly preeclampsia, complicates 10 million pregnancies, resulting in 76,000 maternal mortality and the loss of 500,000 fetal and/or newborns worldwide (Khowaja et al., 2015). Nearly all of these maternal deaths (>99%) occur in low-and middle-income countries (Duley, 2009). In addition, preeclampsia is a risk factor for cardiovascular disease for both mother and child later in life (Agatisa et al., 2004; Mongraw-Chaffin et al., 2010; Davis et al., 2012a). Women who have had preeclampsia exhibit at least a two-fold increased risk of stroke, while risk of death due to ischemic heart disease is eight times higher when preeclampsia occurs before 34 weeks of gestation (Mongraw-Chaffin et al., 2010). Indeed, the American Heart Association (AHA) has included preeclampsia as a risk factor for future cardiovascular disease (CVD) with the recommendation to obtain a history of preeclampsia and to improve lifestyle behaviors of women with such a history (Agatisa et al., 2004; Seely et al., 2013).

Preeclampsia is also a major cause of infant morbidity and mortality worldwide (Xiong et al., 2002; Duley, 2009). Stillbirth is more common in preeclamptic pregnancies while one third of infants of preeclamptic women are growth restricted (Sibai et al., 2005; Villar et al., 2006) and preterm delivery is twice as common in preeclampsia as in normotensive pregnancies (Villar et al., 2006). Furthermore, numerous epidemiological and experimental studies suggest an important role for an adverse intrauterine environment in the development of chronic disease in adult life (Hanson and Gluckman, 2008; Glover, 2011; Davis et al., 2012b). Applying this concept to preeclampsia, many epidemiological studies (Kajantie et al., 2009; Wu et al., 2009, 2011; Davis et al., 2012a,b; Lawlor et al., 2012) indicate that preeclampsia is associated with long-term adverse outcomes in the offspring. The majority of studies report that children and adolescents who were exposed to preeclampsia or hypertension in pregnancy exhibit higher systolic and diastolic blood pressure compared with non-exposed children or adolescents (see details in Davis et al., 2012a). In addition, Kajantie et al. (2009) reported that the risk for stroke in subjects born from preeclamptic pregnancies was twice that of controls born from normotensive pregnancies. Other studies have described an increased risk for pulmonary hypertension (Jayet et al., 2010), metabolic and endocrine disease (Wu et al., 2009, 2011), depression (Tuovinen et al., 2010), cerebral palsy (Szymonowicz and Yu, 1987), poor cognitive outcome (Cheng et al., 2004), or intellectual disabilities (Griffith et al., 2011) in children born of preeclamptic pregnancies compared to non-exposed children.

## Pathophysiology of preeclampsia

Preeclampsia is characterized by impaired cytotrophoblast transformation toward extravillous trophoblasts that result in reduced invasion into the maternal vascular bed (Burton et al., 2009a,b). This phenomenon leads to reduced trophoblastic invasion into maternal spiral vessels preventing their transformation into capacitance vessels. This in turn impairs maternal blood flow to the placenta and also results in high perfusion pressure in the intervillus space generating shear stress to the trophoblast (Burton et al., 2009b). This stress, damages trophoblast leading to detachment and release of cell fragments, microparticles, and extracellular vesicles (EVs; including a wide range of size, see below) into the maternal circulation (Tannetta et al., 2013). Within these EVs harmful elements can be transported into maternal circulation causing maternal endothelial dysfunction. At the same time, these changes generate a vicious cycle that also affects the placental blood flow leading to further release of placental materials that adversely affect maternal endothelial function (Roberts and Escudero, 2012). Not surprisingly, harmful molecules from the placenta can also reach the fetal circulation causing endothelial dysfunction. Indeed, many reports including some from our group (Wadsack et al., 2012; Escudero et al., 2014a) have described feto-placental endothelial dysfunction accompanying preeclamptic pregnancies.

Amongst other harmful molecules released from the placenta, the soluble vascular endothelial growth factor receptor type 1 (sFlt1) has received much attention in preeclampsia. However, many other factors are also involved in the harmful signaling causing endothelial dysfunction in the maternal circulation. Some of the most recently identified elements are placental exosomes, containing molecules such as microRNAs that can incorporate into the maternal cells and modify the expression of targets genes. Currently, study of EVs and microRNA in the maternal circulation and/or in placentae from preeclampsia is extensively studied (see for instance Chen and Wang, 2013; Fu et al., 2013a), since potential predictive tools and mechanistic insights can be obtained using microRNA-omic analysis.

## Endothelial dysfunction in preeclampsia

Endothelial dysfunction is a systemic pathological state characterized by an imbalance between vasodilator and vasoconstrictor molecules produced by or acting on the endothelium (Brunner et al., 2005; Deanfield et al., 2005). In this regard, several publications have described endothelial dysfunction in maternal (Rodgers et al., 1988; Roberts et al., 1989; Roberts, 1998), and in the feto-placental circulation during preeclamptic pregnancies (see details in Sobrevia et al., 2012; Wadsack et al., 2012) or in children born to women with preeclampsia (Jayet et al., 2010; Lazdam et al., 2010; Kvehaugen et al., 2011; Davis et al., 2012b).

Amongst the many markers associated with impaired endothelial function, we will highlight those related to angiogenesis (Shibuya, 2006; Escudero et al., 2009) and metabolic regulation of endothelium (Verdegem et al., 2014). sFlt-1 is a decoy receptor for the vascular endothelial growth factor (VEGF) which binds VEGF and placental growth factor (PlGF) to prevent activation of VEGF receptor type 2 (VEGFR2) in mothers (Parra et al., 2005; Chaiworapongsa et al., 2008, 2010, 2011; Kusanovic et al., 2009; Veas et al., 2011; Parra-Cordero et al., 2013), and feto-placental tissues (Bosco et al., 2012; Escudero et al., 2013, 2014b). High levels are present in preeclamptic pregnancies. There is extensive evidence indicating that VEGF/VEGFRs dysregulation is present in preeclampsia as a hallmark of endothelial dysfunction in mothers and perhaps in their children. Another indicator of endothelial dysfunction is the impaired synthesis and bioavailability of nitric oxide (NO), a vasodilator and angiogenic regulator. This is due in large part to reduced expression and/or activation of endothelial nitric oxide synthase (eNOS) and/or inactivation of NO by reactive oxygen species associated with imbalance between oxidase activity and antioxidant enzyme systems (Myatt and Webster, 2009) or competitive inhibition by asymmetric dimethylarginine (ADMA; Speer et al., 2008).

Another example of endothelial dysfunction in preeclampsia is related to transport and catabolism of metabolic active substrates, including glucose, amino acids, or fatty acids. This is a relevant issue since most of the energy of endothelial cells comes from glycolysis (Verdegem et al., 2014). In preeclampsia, inactivation of glucose-6-phosphate dehydrogenase (G6PD), a rate-limiting enzyme in glucose metabolism, occurs in the fetal circulation, a phenomenon associated with the vascular dysfunction and oxidative stress observed in this disease (Afzal-Ahmed et al., 2007). Similarly, reduced transport and/or metabolism of other bioactive molecules such as adenosine or L-arginine (Casanello et al., 2007), or metabolism of other sources of energy such as fatty acids (Wadhwani et al., 2014), might also contribute to the metabolic alterations leading to endothelial dysfunction in preeclampsia.

It has been proposed that cell fragments observed in maternal circulation during normal or pathological pregnancies are mechanisms for signaling between the fetus and the mother. Feto-maternal signaling may induce vascular and cardiac adaptations during normal pregnancies, which could be pathologically exaggerated during preeclampsia. At least two mechanisms of signaling will be described in this manuscript that are mediated by exosomes and microRNAs. Dozens of studies have sought to decipher their role in signaling between fetus and mother, or as biomarkers of preeclampsia. However, few have studied the endothelial cell as a potential target for exosomes and miRNAs released from the placenta.

## Extracellular vesicles

Extracellular vesicles (EV) are lipid-bilayer structures that are released from cells into the extracellular environment (Mitchell et al., 2015). They contain proteins, miRNA, growth and apoptotic factors, and other regulatory components to induce cell-to-cell communication and signaling throughout the body (Colombo et al., 2014). EVs are released under normal and pathological conditions. It is apparent that multiple EV types can be produced from different cells, including red blood cells (Simpson et al., 2008), fibroblasts (Stadtman and Levine, 2000), endothelial cells (Winyard et al., 2011), and trophoblasts (Salomon et al., 2013). After secretion from cells, the EVs may modify the activity of adjacent cells or travel to regions distal to the site of release in several bodily fluids (e.g., lymph, saliva, blood, mammary glands secretions; Yanez-Mo et al., 2015). EVs are distinguished by size, function, biogenesis (Cocucci et al., 2009), and morphology (Mathivanan et al., 2010) into three categories: microvesicles, apoptotic bodies, and exosomes. Please review Mitchell et al. (2015).

Exosomes are the smallest of EVs, 40 to 120 nm. They are characterized by their endosomal origin and formation through the inward budding of multivesicular bodies (MVB; Kowal et al., 2014). Their density ranges from 1.13 to 1.19 g/mL and they are released into biofluid compartments via exocytosis (Kowal et al., 2014). Exosomes are enriched with endosomal membrane markers (e.g., CD9, CD63, TSG101, and CD81; Mitchell et al., 2015).

Microvesicles, or microparticles, are larger than exosomes, 100–1000 nm in size (Akers et al., 2013). They are the products of tumors (Whiteside, 2005), erythrocytes (Aatonen et al., 2014), and platelets (Fourcade et al., 1995). They are classified as ectosomes due to their derivation from the plasma membrane (Heijnen et al., 1999) and their biogenesis has been shown to be controlled by key proteins such as ARF6 (D'souza-Schorey and Chavrier, 2006), RhoA (Li et al., 2012), and Calpain (Crespin et al., 2009). Microvesicles are enriched with CD40 protein markers (Kowal et al., 2014).

Apoptotic bodies are formed by direct budding of cells during apoptosis and are the largest EV, 1000–5000 nm in size (Perez-Hernandez and Cortes, 2015). They also enriched with histones and DNA (Kowal et al., 2014). Since the size range of the several vesicles overlap, isolation, and characterization remains a challenge in EV research. Nonetheless, EV content is cell-specific and has distinct functions directed at specific cells (Mathivanan and Simpson, 2009). No universal EV isolation technique has been established and consequently many strategies are used for the separation of these vesicles. These include differential density ultracentrifugation, exosome growth enrichment, immunoaffinity beads directed toward surface proteins, and size-exclusion chromatography. Despite that, characterization of size, cargo, and function of EVs and particularly exosomes is one of the cutting-edge topics in preeclampsia (Mitchell et al., 2015).

In addition, the release of EVs is dependent on their microenvironment, influenced by factors such as oxygen tension or glucose concentrations. Glucose regulates intracellular Ca^2+^ concentration, and via this mechanism may alter the rate of exocytosis. At high D-glucose concentrations, release of exosomes from trophoblast cells is increased (Rice et al., 2015) either by an increased rate of exocytosis, the migration of multivesicular bodies (MVBs) to the plasma membrane, increased EV production within MVBs, or a combination of these factors (Savina et al., 2003; MacDonald et al., 2005; Dai et al., 2011). Oxygen concentration also plays a key role in EV release and content regulation.

Incorporation of EVs into recipient cells, and in particular into endothelial cells, involves toll like receptor (TIR) signaling. Thus, endothelial cells incubated with EVs derived from endothelium that have been stimulated by anti-β2 glycoprotein, a phospholipid-binding protein linked with endothelial dysfunction and present in the anti-phospholipid syndrome, exhibited increased phosphorylation of IRAK4, a downstream protein in the TIR signaling pathway (Wu et al., 2015). In other experiments IRAK siRNA inhibited EV-induced endothelium activation, as measured by increased E-selectin cell surface expression. In order to determine which toll like receptor (TLR) may be involved in this phenomenon, investigators used siRNAs for TLR2, TLR4, TLR7, and TLR9. Only treatment with TLR7 and TLR9, blocked EV-induced endothelium activation. TLR9 siRNA also decreased expression of TLR7, suggesting TLR7 as the main regulator of EVs effect on endothelial cells. Since TLR7 is activated in response to ssRNA and miRNAs, authors performed experiments in presence of RNase A, which also inhibited activation of endothelial cells. Finally, EVs from endothelium stimulated by anti-β2 glycoprotein had at least 12 miRNAs upregulated and six downregulated compared to unstimulated control cells. miR126, was identified as targeting endothelium. These results indicate a paracrine pathway for EVs-mediated activation of endothelial cells, with selective incorporation of EVs into target cells directed by TLR7.

## Extracellular vesicle function in pregnancy

**Figure 1:**
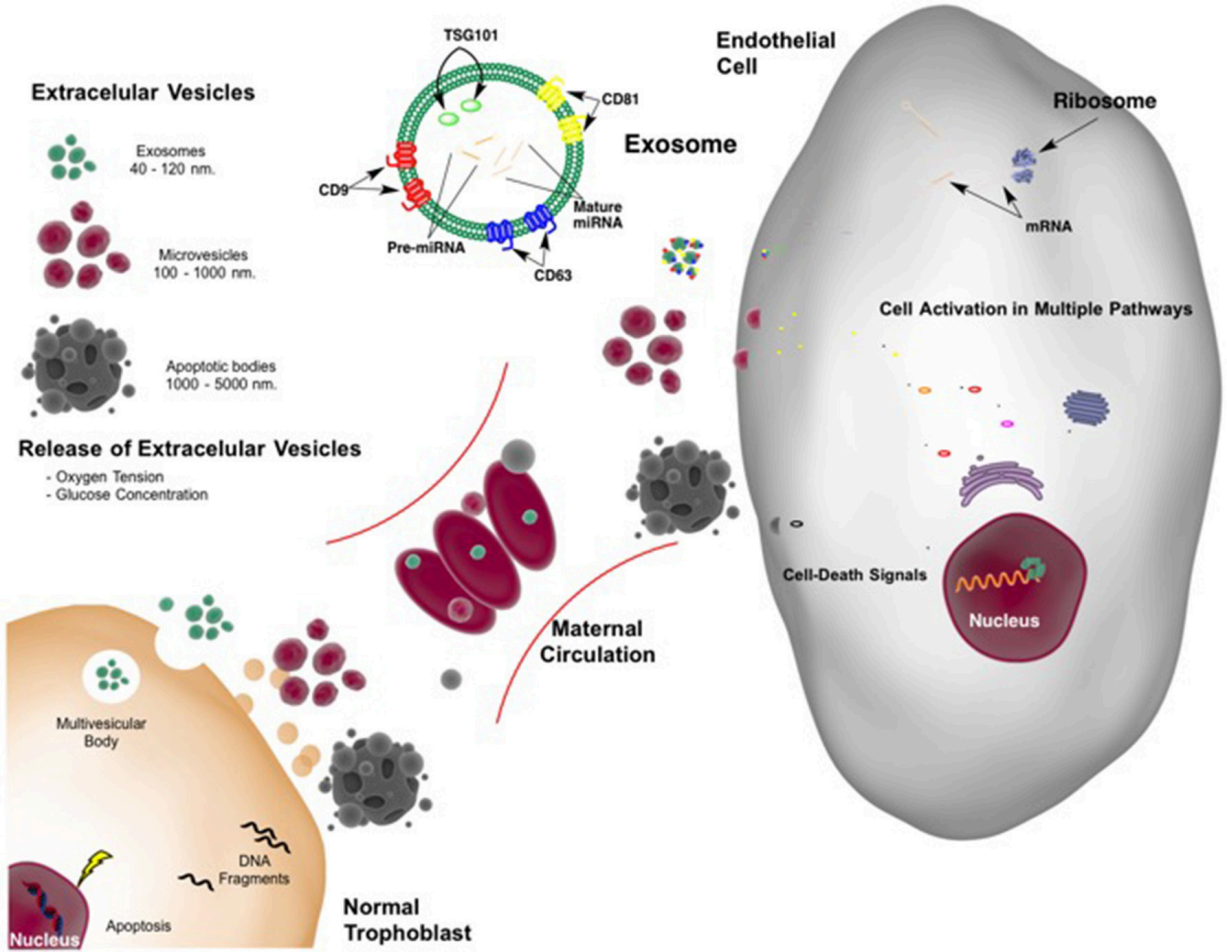
**Extracellular vesicles released from placenta in normal pregnancy**. Extracellular vesicles (EVs) include exosomes, microvesicles, and apoptotic bodies all with different size and origin. With adequate blood flow from the mother to the placenta resulting in normal oxygen tension and glucose (nutrient) concentration, a limited number of EVs are shed from the placenta into the maternal circulation. Cargo and function of the EVs are not completely understood. However, they may constitute a common language between feto-placental tissue and mother with interchange of information leading to normal blood flow supply (from the mother to the placenta). Feto-placental extracellular vesicles may also modulate maternal endothelial cell function. The cargo of exosomes, of endosomal origin, including proteins and nucleic acids, such as microRNAs may be “planned” by the placenta. This cargo controls endothelial cell protein expression leading modifying multiple pathways including among others metabolic and survival-death signals. Appropriate communication allows successful pregnancy and fetal development.

The concentration of placentally derived exosomes in maternal plasma increases progressively (see Figure 1; Sarker et al., 2014). It has been suggested that EVs may be active mediators that communicate between the maternal endometrium and the embryo (Ng et al., 2013) at the time of implantation regulating endometrial remodeling. They are also proposed to regulate physiological adaptation throughout pregnancy (Ng et al., 2013; Sarker et al., 2014), including the modification of maternal immune cell responses and syncitiotrophoblast function locally and systemically. For example, under hypoxic conditions during early pregnancy, cytotrophoblast cells and placental mesenchymal stem cells are stimulated to increase EV release, accompanied by changes in vesicle contents and bioactivity (Salomon et al., 2013).

EVs have also been linked to spiral artery (SpA) remodeling, an adaptation necessary to provide sufficient gas and nutrient exchange from the maternal to fetal side of the placenta (Harris, 2011). The result of normal SpA remodeling is the formation of maternal arteries that are dilated and with reduced resistance to flow of blood from mother to the intervillus space (Cockell and Poston, 1997). This remodeling is associated with extravillous EV signaling and is proposed to stimulate endothelial cell migration resulting in vascular tube formation (Zhang H. C. et al., 2012). Interestingly, placental-derived exosomes carry syncytin proteins, which contribute to fusion of cytotrophoblast cells to form syncytiotrophoblast cells, which in turn constitute the maternal-placental interface (Record, 2014; Vargas et al., 2014).

Extracellular vesicles may also induce release of proinflammatory cytokines (Atay et al., 2011). The interaction between EV proteins and peripheral blood mononuclear cells or dendritic cells can initiate stem cell differentiation. This promotes increased cell migration and suppresses the activation of natural killer cells and macrophages altering inflammatory response during pregnancy (Mincheva-Nilsson et al., 2006; Knight, 2008). Exosomes secreted from placenta contain specific proteins and act upon target immune cells to provide an immunosuppressive environment during pregnancy. Proteins isolated from trophoblast cells suppress maternal immune system, which is essential for fetal semiallograft survival. MHC class I related molecules (Mincheva-Nilsson et al., 2006), down regulated Natural killer cell receptor (NKG2D; Hedlund et al., 2009), functional Fas ligand (Frangsmyr et al., 2005), and TRAIL molecules, the HLA-G and B7 family of immunomodulators (Kshirsagar et al., 2012) isolated from first trimester placental tissues suppressed T cell signaling components.

## Extracellular vesicles in preeclampsia

**Figure 2:**
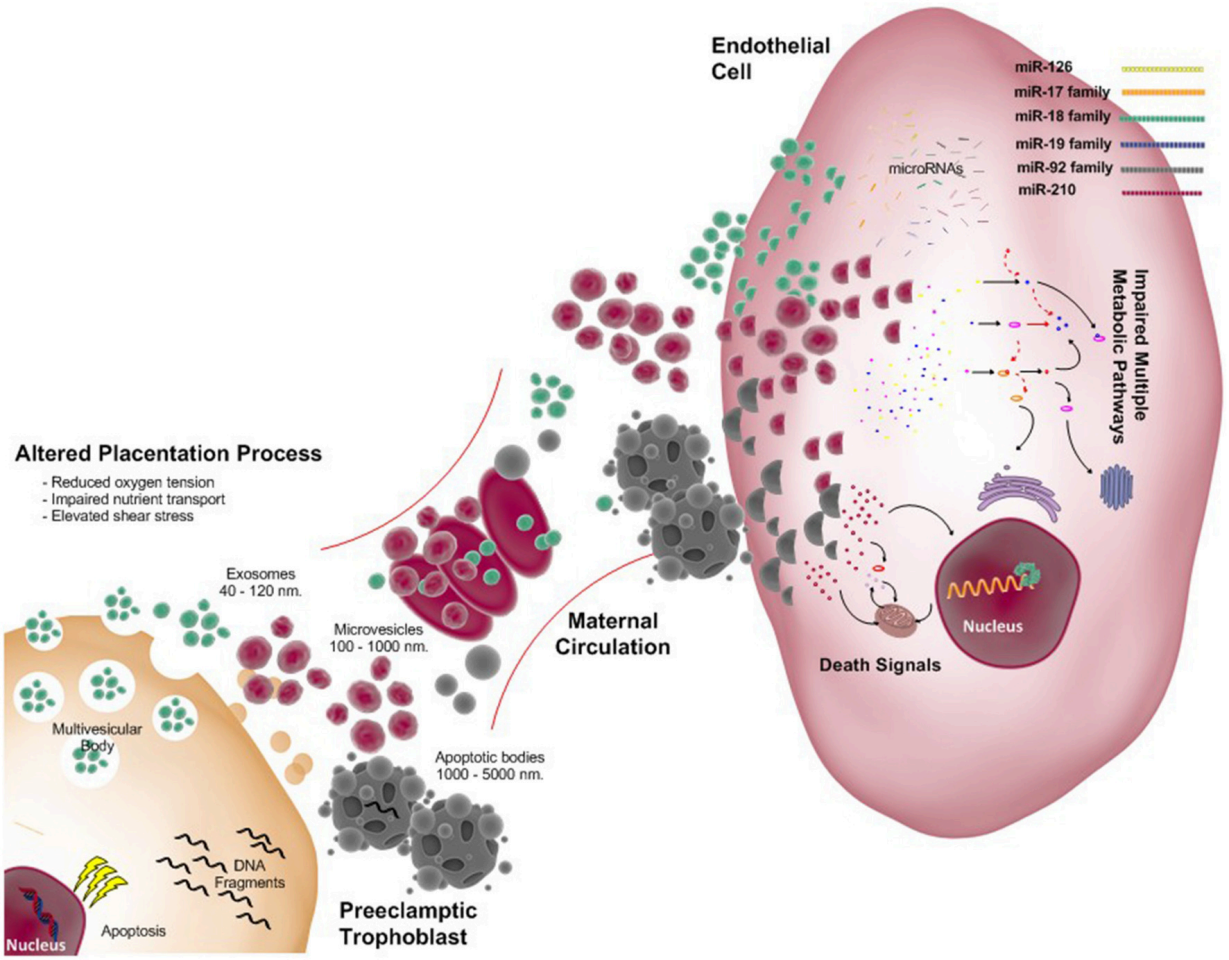
**Exosomes released from the placenta: focus on preeclampsia**. As in normal pregnancy extracellular vesicles (EVs) include exosomes, microvesicles, and apoptotic bodies all with different size and origin are present. However, abnormal placentation secondary to reduced trophoblast invasion and failed remodeling of spiral arteries leads to reduced oxygen tension, impaired nutrient transport and exposure to increased shear stress for the syncitiotrophoblast. Under these stressful conditions more EVs are shed from the placenta into the maternal circulation. Messages carried by in the EVs are not completely understood; however, it is proposed that they cause endothelial dysfunction with associated elevation of maternal blood pressure. This figure indicates some of miscommunication generated by the placenta via exosomes and its cargo, microRNAs. Potential effects upon maternal endothelial cells by the family of miR-126, miR-17, miR-18, miR-19, miR-92, and miR-210 are detailed in the manuscript. In preeclampsia multiple pathways may be impaired including metabolic and death signaling by this microRNAs among others.

In preeclampsia, impaired placental function with placental apoptosis and necrosis causes increased release of microvesicles and nanovesicles (i.e., exosomes; see Figure 2). These exosomes contain proteins, miRNA, DNA, RNA; as well the lipids comprising the vesicular wall. These components are involved in several stages of the pathogenesis of preeclampsia. Extracellular vesicles (including exosomes) originating from placental explant and placental cells promote pro-inflammatory cytokines production (Germain et al., 2007) and endothelial dysfunction (Cockell et al., 1997).

Using a placental marker (i.e., Placental Alkaline Phosphatase, PLAP), placenta-derived vesicles were identified in maternal plasma as early as the first trimester of pregnancy (Cockell et al., 1997). The immunoreactive exosomal PLAP was not detectable in plasma of non-pregnant women (Sabapatha et al., 2006; Dragovic et al., 2013; Salomon et al., 2014a). The role of placental exosomal bioactivity (i.e., release, content and interaction with target cells) under normal or pathological conditions (e.g., PE) has not been fully established (Mitchell et al., 2015). Nonetheless, some models have been used to address this issue. For instance, with hypoxia, the number of extracellular vesicles released from several cell types are modified (Salomon et al., 2013). At lower oxygen concentrations, the amount of functional EVs increases leading to a spike in EVT migration. The content of the EVs are also changed, which may cause endothelial cell dysfunction and direct placentation toward abnormal SpA remodeling.

Specific protein and lipids of EVs produced by syncytiotrophoblasts are altered in preeclampsia (Redman and Sargent, 2008). For instance, among 400 proteins identified within syncytiotrophoblast-derived EVs, at least 25 of these were unique to preeclamptic pregnancies, including integrins, annexins, and histones (Baig et al., 2014). In another study (Vargas et al., 2014) the specific syncytiotrophoblast protein, syncytin-2, was markedly down-regulated in exosomes from placentas of pregnant women with preeclampsia compared to normal pregnancies. In addition, the EV lipid profile in human placental syncytiotrophoblast microvesicles derived from preeclamptic pregnancies identified ~200 distinct lipids. These included a higher concentration of phosphatidylserine and a lower concentration of phosphatidic acid, ganglioside mannoside 3, and phosphatidulglycerol (Baig et al., 2013).

MicroRNAs are also present within exosomes (see section below). Placental specific miRNA molecules were identified within placental exosomes (Ouyang et al., 2014) that were resistant to viral infection apparently to protect the fetus during pregnancy (Bullerdiek et al., 2013; Delorme-Axford et al., 2013; Mouillet et al., 2014). Exosomes are released in low concentration during normal pregnancy but release from syncitiotrophoblast is increased in endothelial and immune-cell dysfunction as is associated with the placental pathophysiology in preeclampsia (Pant et al., 2012).

In view of this evidence, it is hypothesized that the effect of these nanovesicles is determined by the cellular origin and/or exosomal content (e.g., proteins, miRNA, DNA, RNA, lipids, etc.). These are proposed to direct placental function in early normal pregnancy but also to be potentially important to disorder placentation in complicated pregnancies (Salomon et al., 2014a) providing a potential biomarker for preeclampsia (see Table 1).

**Table 1:** **Summary of studies of exosomes and microparticles in preeclampsia**.

**Extracellular vesicle population**	**Subcellular origin**	**Size, nm**	**Alteration in preeclampsia**	**References**
Exosomes	Endocytic pathway	40–120	Syncytin-2 lower in placental exosomes	Vargas et al., 2014
			63 proteins uniquely present in CTB-binding vesicles, and 80 in AV-binding vesicles in preeclamptic samples. No description of main biological pathways	Tan et al., 2014
			Total exosomes concentration and placenta-derived exosomes were elevated in plasma at first trimester of pregnancy (i.e., 11–14 weeks) in women who develop PE later in pregnancy	Salomon et al., 2014b
			29 proteins associated with multiple biological functions including complement and coagulation cascade were differentially expressed in umbilical blood exosomes	Jia et al., 2015
			Exosomes positive for AQP2 were isolated from urine obtained from patients with PE	Nielsen et al., 2015
Microvesicles or shedding vesicles	Plasma membrane	50–1000	Uncharecterized microvesicles higher in plasma	Dragovic et al., 2013
			Elevated tissue factor within STBM	Gardiner et al., 2011
			Elevated STBM in early and late onset preeclampsia	Chen et al., 2012
			Elevated levels of DNA-associated placental microparticles	Orozco et al., 2009
			Alteration in lipids including higher PPTS and lower PPA, PPDG, and GM3.	Baig et al., 2013
			Proteomic analysis of STBM. Major biological functions altered: cell death and survival, cellular assembly and organization, immune response, lipid metabolism, and carbohydrate metabolism	Baig et al., 2014

PPTS, Phosphatidylserine; PPA, phosphatidic acid; PPDG, phosphatidylglycerol; GM3 ganglioside mannoside 3; STBM, syncytiotrophoblast microvesicles; CTB, Cholera toxin B chain; AV, Annexin V; AQP2, Aquaporin-2.

## Overview of microRNA

Micro-RNAs (miRNAs, 21–25 nucleotides) are critical regulators of gene expression. Canonically, miRNAs are transcribed by RNA polymerase II from individual miRNA genes, intron of protein coding genes, or polycistronic transcripts such as capped and polyadenylated primary miRNA transcripts (pri-miRNA, ~70 nucleotides). Several nuclear and cytoplasmic enzymes process pri-miRNAs, and miRNA are finally processed to 22 nucleotides by the cytoplasmic enzyme, Dicer. Following processing, the miRNAs are incorporated into the RNA-induced silencing complex (RISC) that mediates miRNA binding to the 3′ untranslated regions (3′UTR) of targeted messenger RNA (mRNA). This binding negatively regulates gene expression by translation inhibition, mRNA degradation or a combination of both (Chamorro-Jorganes et al., 2013; Araldi et al., 2015). In order to repress transcription, a crucial sequence of miRNA located in position 2–8 (i.e., seed sequence) must be almost perfectly complimentary to regions at the 3′UTR of the targeted genes. Since, computational analysis indicates that 60% of protein-coding genes harbor miRNA targets sites in their 3′UTR (Chen and Wang, 2013; Fu et al., 2013a; Yan et al., 2013), it is likely that a single miRNA could modulate the expression of hundreds of genes (Chamorro-Jorganes et al., 2013; Chen and Wang, 2013; Fu et al., 2013a; Yan et al., 2013; Araldi et al., 2015). The complexity of these regulatory mechanisms is even greater when we consider that miRNA formation includes formation of thermodynamically less stable strands (miRNA^*^), splicing variants, and there is evidence of redundant expression in several genes. Also opposing actions are determined by the targeted cells and their environment (i.e., hypoxia, normoxia, or oxidative stress). There is also cell and tissue specific expression among other regulatory mechanisms (Nishiguchi et al., 2015). Taking all of this into account, it is not surprising that the study of miRNA is one of the hottest topics in the studies of the cell biology of normal and pathological conditions, including preeclampsia. In the latter, there are at least a dozen preeclampsia-associated differentially expressed miRNAs (Chen and Wang, 2013), most of them relevant to impaired trophoblast invasion, that have been used as biomarkers (see Table 1). However, we would like to emphasize that few of these studies are related to the study of endothelial function.

## Exosomes, microRNA, and endothelial regulation: focus on angiogenesis

Exosomes as a mechanism of intercellular communication influence many endothelial functions, including vascular tone, interaction between endothelial cells, smooth muscle cells and pericytes, and angiogenesis. Also, a number of studies have demonstrated a correlation between the number of circulating (CD31^+^ CD41^−^) microvesicles and endothelial dysfunction in coronary artery disease, type 2 diabetes mellitus, hypertension, atherosclerosis, or cancer. miRNAs in exosomes have a potential beneficial effects and may be involved in the pathogenesis of cardiovascular diseases. There are numerous reports demonstrating the effect of exosomes on endothelial function. In this manuscript we will summarize such reports of exosomes, miRNAs, and angiogenesis but interested readers may find additional relevant information in other excellent reviews (Chistiakov et al., 2015; Das and Halushka, 2015; Lawson et al., 2016).

Halkein et al. (2013) studied peripartum cardiomyopathy (PPCM) a life-threatening pregnancy-associated heart dysfunction in previously healthy women. They found that the 16-kDa N-terminal prolactin fragment, a peptide linked with occurrence with PPCM, stimulated the release of miR-146a-loaded exosomes from endothelial cells. Their target cells were cardiomyocytes, which after incorporation of these “mir-146a-loaded exosomes” decreased the expression of genes including Erbb4, Notch 1, and Irak1 and down regulating metabolic activity of cardiomyocytes. Further *in vitro* and *in vivo* experiments, confirmed the involvement of miR-146a in the PPCM. miRNAs contend in exosomes which target are endothelial cells may down or up regulated angiogenesis process. In the following section we present relevant studies illustrating exosomal miRNAs that target endothelial cells and in particular affect angiogenesis (see Table 2).

**Table 2:** **miRNAs incorporated into exosomes that targeted endothelial cells modulating angiogenesis**.

**miRNA**	**miRNA source**	**Recipient cell**	**Target gene**	**References**
miR-21	Human lung cancer	HBEC	VEGF	Liu et al., 2016
miR-17, miR-20a, miR023a, miR-23b, miR-30b, miR-30c, miR-126-3p, miR-132	Mice cardiomyocytes	HUVEC	Multiple target gene	Garcia et al., 2015
miR-23, miR-320b	MCF7	EA.hy926	PLAU, AMOTL1, NRP1, ETS2	Hannafon et al., 2015
miR-126	HUVEC	HUVEC	SPRED1, VECAM1, RGS16, CXCL12	Zernecke et al., 2009
miR-132	CPC	HUVEC	RasGAp-p120	Barile et al., 2014
miR-135b	HR-MM	HUVEC	FIH-1	Umezu et al., 2014
miR-143	PASMC	PAEC		Deng et al., 2015
miR-150	THP-1	HMEC-1	c-Myb	Zhang et al., 2010
miR-320	Cardiomyocytes	CEC	IGF1, HSP20, Ets2	Wang X. et al., 2014
miR142-3p, miR-223-3p	BMDM	iECL	Multiple miRNAs	Squadrito et al., 2014

HBEC, Human bronchial epithelial cells; HUVEC, human umbilical endothelial cells; CPC, cardiac progenitor cells; CEC, cardiac endothelial cells; PASMC, Pulmonary artery smooth muscle cells; PAEC, pulmonary aortic endothelial cells; HR-MM, hypoxia resistant myelomas cell lines (RPMI8226); BMDM, Bone marrow derived macrophages; iECL, immortalized endothelial-like cells from the hearth of Dicer^fl∕fl^ mice.

### miR-21

miR-21 is up regulated in many diseases, in particular cancer, in association with regulation of cell proliferation and apoptosis. The gene for miR-21 is located on chromosome 17q23.2, immediately downstream of the vacuole membrane protein 1 (VMP1) gene. miR-21 is increased in patient who are in high risk of developing lung cancer duo to a smoking habit (Liu et al., 2016). A human bronchial cell lines exposed to smoke extract increase the synthesis and release of miR-21. When exosomes-containing miR-21 isolated from those cells are transferred to endothelial cells they stimulate angiogenesis. Also, when transferred to bronchial cells they increase VEGF in dose-related manner. Exosomes labeled with green fluorescent dye increase incorporation of exosomes-containing miR-21 into endothelial cells. Based upon these observations the authors concluded that exosomes-containing miR-21 may increase tumor angiogenesis in lung cancer.

Exosomes harvested from breast cancer cells exposed to docosahexaenoic acid (DHA) exhibited high levels of 22 miRNAs (see Table 2). miR-21, miR-23b, miR-27b, and miR-320, miRNAs that have known activity in targeting endothelial cells to suppress angiogenesis. Mechanism for anti-angiogenic effect of the last miRNAs was confirmed using either exosomes released by a DHA-treated breast cancer cell line (MCF7 cells) or transfected endothelial cell (i.e., EA.hy926 cells). Each of these miRNAs, upregulated let-7a, miR-21, miR-23b, miR-27b, and miR-320b in the endothelial cells. They also reduced tube formation capacity and the expression of pro-angiogenic target genes (Hannafon et al., 2015; see Table 3).

**Table 3:** **Summary of studies of miRNAs in preeclampsia**.

**miRNAs**	**Levels**	**Target genes**	**References**
**PLACENTA**			
miR-1301, miR-223, and miR-224	↓	Leptin gene	Weedon-Fekjaer et al., 2014
miR-92b, miR-197, miR-342-3p, miR-296-5p, miR-26b, miR-25, miR-296-3p, miR-26a, miR-198, miR-202, miR-191, miR-95, and miR-204	↑	Multiple targets in several signaling pathways, adherent junction, focal adhesion, and regulation of the actin cytoskeleton	Choi et al., 2013
miR-21 and miR-223	↓		
hsa-miR-v5	↓	Not reported	Lazar et al., 2012
miR-17, -20a, and -20b	↑	EPHB4 and Eph-B2	Wang W. et al., 2012
miR-20a, miR-210, miR-451, miR-518c, and miR-526b^*^	↑	HSD17B1	Ishibashi et al., 2012
**PLACENTA AND TROPHOBLAST**			
miR-125b-1-3p	↑	S1PR1	Li Q. et al., 2014^†^
miR-210	↑	KCMF1	Luo et al., 2014^†^
miR-210	↑	Iron sulfur cluster	Muralimanoharan et al., 2012
miR-210	↑	ERK signaling	Anton et al., 2013^†^^α^
‘miR-18a, miR-19b1, and miR-92a1	↓	Smad2 (miR-18a)	Xu et al., 2014
miR-210	↑		
miR-155	↑	eNOS	Li X. et al., 2014^†^
pri-miR-34a	↑	SERPINA3	Doridot et al., 2014
miR-101	↓	ERp44	Zou et al., 2014^‡^
miR-195	↓	ActRIIA	Bai et al., 2012
miR-106a and −19b	↑	hCYP19A gene and hGCM1	Kumar et al., 2013^†^
**TROPHOBLAST CELL LINES**			
miR-376c	↓	ALK5 and ALK7	Fu et al., 2013b
miR-20a	↑	FOXA1	Wang Y. et al., 2014
miR-29b	↑	MCL1, MMP2, VEGFA and ITGB1	Li H. et al., 2013
**STEM CELLS**			
microRNA-494	↑	CDK6, CCND1, VEGF	Chen et al., 2015
miR-136, miR-495, miR-16, miR-29b and miR-494	↑	Multiple targets involved in angiogenesis (VEGF), inflammation, differentiation of MSC	Zhao et al., 2014
miR-126	↓	PIK3R2	Yan et al., 2013
miR-16	↑	CCNE1, VEGF	Wang Y. et al., 2012
**PLASMA AND WHOLE BLOOD**			
miR-1233	↑	Not reported	Ura et al., 2014
C19MC microRNAs (miR-516-5p, miR-517^*^, miR-520a, miR-520h, miR-525, and miR-526a)	↑	Not reported	Hromadnikova et al., 2013
miR-141, miR-144, miR-221, and miR-29a	↑	Not reported	Li et al., 2013
miR-516-5p, miR-517^*^, miR-518b, miR-520a^*^, miR-520h, miR-525, and miR-526a	↑	Not reported	Hromadnikova et al., 2012
miR-24, miR-26a, miR-103, miR-130b, miR-181a, miR-342-3p, and miR-574-5p	↑	Not reported	Wu et al., 2012

Data since 2012. CDK6, Cyclin-dependent kinase 6; CCND1, Cyclin D1; VEGF, Vascular endothelial growth factor; S1PR1, Sphingosine-1-phosphate receptor 1; FOXA1, Forkhead box protein A1; KCMF1, Potassium channel modulatory factor 1; eNOS, endothelial nitric oxide synthase; ERp44, Endoplasmic reticulum protein 44; Smad2, SMAD family member 2; SERPINA3, Serpin peptidase inhibitor, clade A (alpha-1 antiproteinase, antitrypsin); member 3, ERK, Extracellular-signal-regulated kinases; hCYP19A, Human aromatese gene; GCM1, transcription factor glial cells missing 1; PIK3R2, phosphoinositide-3-kinase regulatory subunit 2; ALK5 and ALK7, Activin receptor-like kinase 5 and ALK7; CCNE1, Cyclin E1; ActRIIA, Type II receptor for Activin A and Nodal; MCL1, myeloid cell leukemia sequence 1; MMP2, matrix metallproteinase 2; ITGB1, integrin β1; EPHB4, EPH receptor B4; EphB2, Ephrin-B2; HSD17B1, hydroxysteroid (17-β) dehydrogenase 1;

† , Report reduce trophoblast invasion;

‡ , Enhance trophoblast apoptosis;

α , Predictive role for preeclampsia;

* *, From the opposite arm of the precursor*.

In placentas from preeclamptic pregnancies, down regulation (Choi et al., 2013), or up regulation of miR-21 in placentas with abnormal Doppler analysis has been reported, where there were no changes with preeclamptic women with normal Doppler (Cindrova-Davies et al., 2013). Although, no specific target was analyzed in this last study, bioinformatics reveals potential target for miR-21 such as nuclear factor I/B (NFIB) and ras homolog family member B (RHOB) genes (Choi et al., 2013).

Others have studied cardiomyocytes-endothelial crosstalk by miRNAs containing in exosomes. Garcia et al. (2015) studying cardiomyocytes isolated from neonatal mice and exposed to low glucose medium (starvation conditions), found that amongst the 380 miRNA tested, 30 miRNA were expressed in starvation, 13 miRNA in control, and only eight miRNA in both conditions. in these were significantly increased with starvation. The main groups of genes were associated with cell proliferation, cell-cycle, and protein transport. Target genes included MAPK, Wnt, p53, VEGF, Notch signaling pathway, endocytosis, among others. Interestingly for our current analysis, when exosomes from cardiomyocytes were transferred to endothelial cells, they were incorporated, this was associated with up-regulation of pro-angiogenic related genes such as angiopoietins type 1 (ANGPT) and angiopoietin-like protein 4 (ANGPTL4). Accordingly, cardiomyocytes-derived exosomes increase endothelial proliferation and tube formation capacity. The study suggests that miRNA within exosomes can modulate cell–cell communication. However, which miRNA might direct these effects upon pro-angiogenic endothelial capacity was not specified. In the heart, there is also cross talk between the cardiac progenitor cell (CPC) population and endothelial cells. An example of this is found in the work by Barile et al. (2014) who characterized exosomes released by CPC, which content include several angiogenic related miRNA, miR-210, miR-132, and miR-1461-3p. A cardioprotective role of miR-210 and miR-132 was further characterized, with evidence that both induce apoptotic protection of a cardiomyocytic cell line (HL-1). In addition, miR-132 promoted the formation of endothelial tubes, confirming the pro-angiogenic role of miRNAs packaged into exosomes. This has been reviewed by Cervio et al. (2015).

Other examples include pulmonary artery smooth muscle cells (PASMCs) cross talk with endothelial cells. Deng et al. (2015) observed high abundance of miR-143-3p in PASMC-derived exosomes. Similar to the previous description, a paracrine pro-angiogenic effect of miR-143-3p-enriched exosomes from PASMC transferred to pulmonary arterial endothelial cells. No specific target for miR-143-3p was identified in this study. Interestingly, a deficiency of miR-143 prevented the development of pulmonary hypertension in an *in vivo* experiment (Deng et al., 2015).

## Angiogenesis as a target of miRNA in preeclampsia

The relevance of miRNA to endothelial function is demonstrated by knockdown of Dicer in endothelial cells, which inhibits proliferation and tube formation *in vitro* (Kuehbacher et al., 2007). Endothelium-specific Dicer knockout mice have impaired blood vessel development (Suarez et al., 2007). Furthermore hypoxia, a phenomenon present in preeclampsia, down-regulates Dicer function, and expression in endothelial cells (Ho et al., 2012). An increasing number of publications have investigated endothelial-related miRNAs and their potential importance to preeclampsia. Most of them, however, have focused on the placenta or maternal blood (see Table 3). In the next section we describe miRNAs that target endothelial cells. There is, however, no information as to whether these miRNAs are present in exosomes.

### miR-126

miR-126 is encoded by intron 7 of the epidermal growth factor- like domain 7 (egfl7) gene and highly expressed in human endothelial cells (Bai et al., 2014). The precursor pre-miR-126 gives rise to two mature strands, miR-126-3p and miR126-5p. The expression, and target of that miR-126s may be different in endothelial cells (Fish et al., 2008; Wei et al., 2013; Poissonnier et al., 2014). In miR126^−∕−^ mice at least 513 genes (including VEGF, EGFL7, CD31, RGS3, v-CRK) were up regulated in injured carotid arteries isolated from those animals (Schober et al., 2014). Among targets genes, miR-126 negatively regulates sprout-related protein (SPRED1) and phosphoinositol-3 kinase regulatory subunit 2 (PIK3R2/p85-B), which is involved in VEGF pathway (Nishiguchi et al., 2015). Deletion of the gene encoding pre-miR-126 affects vascular integrity and angiogenesis during development without causing overt abnormalities after birth (Fish et al., 2008). However, ischemic neovascularization is severely impaired in miR126^−∕−^ mice (Wang et al., 2008). In preeclampsia, Yang and colleagues reported elevated miR-126 (and mir-126^*^ among other 20 miRNAs) in plasma and placenta (Yang et al., 2015), whereas Yan and colleagues reported decreased miR-126 expression in both umbilical endothelial progenitor cells and placentas from preeclamptic pregnancies (Yan et al., 2013). This latter study found that miR-126 down regulated the expression of the anti-angiogenic gene PIK3R2 that is involved in the negative regulation of PI3K-Akt signaling pathways. Also, miR-126 manifested a pro-vasculogenic capacity, enhancing the proliferation, migration, and angiogenic capacity of umbilical endothelial progenitor cells (ECP). The increased angiogenic capacity was confirmed in pregnant rats in which miR-126 increased vascular sprouting, as well as placenta and fetal weights (Yan et al., 2013). These findings suggest that the reduction in miR-126 present in preeclampsia might impair placental vascular development. In other studies of reduced expression of miR-126 in preeclampsia placentas, there was a correlation of mir-126 levels with placental expression of VEGF (Hong et al., 2014). Consistent with this, in gain-loss assays miR-126 regulated VEGF expression in BeWo cells, confirming that VEGF is a target gene of the miR126. Despite this analysis, comparative expression of miR-126 and its target genes in maternal and fetal circulation during preeclamptic pregnancies has not yet been performed.

### miR17-92 cluster

miR17-92 cluster is one of the best-characterized polysistronic miRNAs. It is located in intron 3 of the C13orf25 gene (chromosome 13q31.3). The cluster contains six miRNAs including miR17, miR-18, miR-19a, miR-19b-1, miR-20a, and miR-92a-1. These are processed from a common precursor transcript. Based on their seed sequences, they are grouped into four families: the miR-17 family (miR-17 and miR-20a), the miR-18 family, the miR-19 family, and the miR-92 family. Based upon sequence analysis miR-17, miR20a, and miR-20b possess overlapping function, targeting similar sets of genes, including hypoxia inducible factor 1 alpha (HIF1A), interleukin 8 (IL-8), tissue inhibitor of metalloproteinases 2, matrix metallopeptidase 2, VEGFA, ephring-B2, and Eph receptor B4 (EPHB4; Wang W. et al., 2012; Chen and Wang, 2013). In endothelial cells, overexpression of miR17-92 cluster was linked to down regulation of anti-angiogenic proteins such as connective tissue growth factor (CTGF), and Tsp-1 (targets of miR-18 and miR-19), and tissue inhibitor of metalloproteinase 1 (TIMP1). This cluster, in particular miR-17 or miR-19, also negatively regulates the expression of pro-angiogenic Janus kinase 1 (JAK1), or cyclin D1 (CCND1; see details in Chamorro-Jorganes et al., 2013). Other target genes down regulated by this cluster, in particular by miR-92a, are sphingosine-1-phosphate receptor 1 (S1P1), mitogen-activated kinase kinase 4 (MKK4), and eNOS (Bonauer et al., 2009). Wang W. et al. (2012) found that miR-17, miR-20a, and miR-20 b were significantly increased in placentas from preeclamptic compared with control placentas. With *in silico* analysis, these authors describe several angiogenesis-related genes, including HIF-1 α-subunit (HIF1A), IL-8, EPHB4, tissue inhibitor of metalloproteinase 2 (TIMP2), VEGF, ephrin-B2 (EFNB2), and matrix metallopeptidase 2 (MMP2) as potential targets of miR-17, miR-20a, and miR-20b. *In vitro* experiments confirm that miR-20b overexpression reduce EPHB4; whereas co-transfection of miR-20b antagomir to inhibit miR-20b expression increased the expression of HIF1A, MMP2, and EFNB2 by about 60–70% in human umbilical vein endothelial cells (HUVEC). Inhibition of miR-20b also reduced VEGFA expression. Altogether these results suggest that decreased expression of cluster miR17-92 might also be related with endothelial dysfunction present in preeclampsia.

### miR-210

mir-210 is a hypoxia-induced miRNAs well-studied in cancer. It is located in the intronic sequence of mRNA transcript *AK123483* that itself is hypoxia-inducible. Multiple groups have found that miR-210 is specifically induced by HIF-1α, and it is considered one of the hallmarks of hypoxic induced response in several cell types including endothelial cells (Chan and Loscalzo, 2010). To date, more than 50 genes have been identified as direct targets of miR-210. These genes are involved in cells processes such as metabolism (mitochondrial proteins, including iron sulfur cluster assembly proteins ISCU1 and ISCU2), cell survival, proliferation and angiogenesis (i.e., fibroblast growth factor receptor, FGFR; ephrin-A3, EFNA3; c-MYC antagonist, MNT; homeobox-A9, HOXA9, etc.; see details in Chan and Loscalzo, 2010). In preeclampsia, miR-210 is one of the miRNAs that consistently (Liu et al., 2012), exhibits high expression in placenta (Pineles et al., 2007; Enquobahrie et al., 2011; Lee et al., 2011; Muralimanoharan et al., 2012; Luo et al., 2014), or increased concentration in maternal whole blood (Gunel et al., 2011; Anton et al., 2013; Murphy et al., 2015) especially in severe preeclampsia (Zhu et al., 2009; Zhang Y. et al., 2012; Xu et al., 2014). Despite this, few studies have investigated miR-210 targeted genes that include potassium channel modulatory factor 1 (KCMF1; Luo et al., 2014), mitochondrial complex III (Muralimanoharan et al., 2012), hydroxysteroid (17-β) dehydrogenase 1 (HSD17B1; Ishibashi et al., 2012), ISCU (Lee et al., 2011), EFNA3 and HOXA9 (Zhang Y. et al., 2012). All of these are associated with reduced trophoblast invasion. Interestingly, no studies have yet been published of the regulation of miR210 in endothelial cells from the maternal or feto-placental circulation.

## Concluding remark and highlighted points

Endothelial dysfunction in both the maternal and feto-placental circulation is a hallmark of preeclamptic pregnancy. This dysfunction includes down-regulation of genes involved in regulation of vascular tone, membrane transporter function, endothelial survival or proliferation, angiogenesis, and metabolic pathways. The question of how these alterations are generated in the preeclamptic pregnancy remains unanswered. There are numerous reports of increased circulating concentration of endothelial-released proteins related to endothelial dysfunction. These are proposed to be released from the placenta. In this scenario, the release of extracellular vesicles from the placenta, has attracted great attention. It is proposed that endothelial function is modified by lipids in the wall of the vesicle but perhaps more importantly by the cargo within the vesicle. Indeed, miRNAs within these vesicles (in particular exosomes) and their target genes may explain the extensive modification of endothelial-protein expression present in preeclampsia. Characterization of the expression of these regulators and their target genes is of great potential importance not only in preeclampsia, but also may indicate artificially constructed cargo exosomes that with appropriate miRNA could constitute a new area of genetic treatment. They also may provide potential biomarkers but more importantly understanding the regulation and role of these molecules should lead to a better understanding of these processes.

The ability of biomarkers to predict women at risk to develop preeclampsia has been extensively studied. However, despite enormous effort, thus far no markers, including exosomes and microRNAs, are effective predictors. This fact is not only due to limitations (i.e., big data, expensive, biological meaning, among others), but also due to the fact that preeclampsia is a syndrome not a discrete disease (Myatt and Roberts, 2015). Currently there are global initiatives searching to characterize subtypes of the disease in different populations, which should facilitate validation of biomarkers. To accomplish this we must consider the epidemiology of preeclampsia. In in low and middle-income countries where there are limitations on resources for expensive technologies, the prevalence and mortality greater than in high-income countries. Thus the clinical application for using exosomes and miRNAs is a huge challenge.

Nevertheless, study of exosomes and its content including miRNAs will allow translation of the language of the remarkable mother-placenta-fetus communication in normal and pathological pregnancies. Exosomes and miRNAs constitute a cell–cell communication system, and we are beginning to understand the relevant mechanisms. We have focused on endothelial cells, and more specifically on angiogenic processes. It is possible that exosomes and miRNAs might have quite different effects depending on the micro-environment. As an example, exosomes released by tumors or with hypoxic stress increase angiogenesis, while in preeclampsia, EVs including exosomes may lead to endothelial dysfunction. Diversity of response is characteristic of EVs. As described in this manuscript, the synthesis of exosomes is modified by hypoxia, glucose content, and pro-oxidative/anti-oxidative balance, among others. This variability is not only in the number of exosomes but also membrane and vesicle content. Recipient cells may also modulate incorporation of these cell particles via TLRs. Once inside recipient cells, cargo is released and message code is read. However, this also will depend upon the micro-environment of the recipient cells. Although, there has been progress in the understanding these processes there is limited information available about pregnancy. Future research should be directed to better understand the role of exosomes and their cargo in mother-placenta-fetal communication. In the field of preeclampsia, there is little information about miRNAs included in exosomes or EVs, that we know have the capacity to modulate gene targets and generate endothelial dysfunction and/or impair angiogenesis. We have summarized information on the actions of miRNA-21, miRNA-126, miRNA-132, miRNA-143, miRNA-210, miRNA-320, and other miRNAs with potential significance to endothelial dysfunction during preeclampsia due to their affect upon angiogenic processes. However, we acknowledge that this constitute a substantial limitation since compared to extensive information on this this topic in the cardiovascular field little is known about preeclampsia. Endothelial dysfunction is a hallmark of preeclampsia and we encourage future research in this area.

At the moment only a few reports have the potential role of endothelial regulator miRNA in preeclampsia. In the few reports available progress has been limited by variability of the analysis including particle size analyzed. Differences in EVs content, and the large number of targets genes including nearly all of those modulating metabolic function in the endothelial cells that might be impaired support the importance of these interactions. Fruitful targets for investigation are underling mechanism determining miRNA release from placenta and their impact on endothelial function.

## Author contributions

This work was carried out as a full collaboration among all the authors. CE defined the research topic. KH, FT, JA, CA prepare draft of the manuscript. JR, GT, GD, GR, and CE edited the text. CE, JR, CS co-wrote the manuscript. All authors approved the final version of the manuscript.

### Conflict of interest statement

The authors declare that the research was conducted in the absence of any commercial or financial relationships that could be construed as a potential conflict of interest.
